# Recognition of depression in people of different cultures: a qualitative study

**DOI:** 10.1186/1471-2296-10-53

**Published:** 2009-07-27

**Authors:** Arja Lehti, Anne Hammarström, Bengt Mattsson

**Affiliations:** 1 Department of Public Health and Clinical Medicine, Family Medicine, Umeå University, 901 85 Umeå, Sweden; 2 Department of Public Health and Community Medicine/Section of Primary Health Care, The Sahlgrenska Academy, University of Gothenburg, Box 454, 405 30 Gothenburg, Sweden

## Abstract

**Background:**

Many minority group patients who attend primary health care are depressed. To identify a depressive state when GPs see patients from other cultures than their own can be difficult because of cultural and gender differences in expressions and problems of communication. The aim of this study was to explore and analyse how GPs think and deliberate when seeing and treating patients from foreign countries who display potential depressive features.

**Methods:**

The data were collected in focus groups and through individual interviews with GPs in northern Sweden and analysed by qualitative content analysis.

**Results:**

In the analysis three themes, based on various categories, emerged; "Realizing the background", "Struggling for clarity" and "Optimizing management". Patients' early life events of importance were often unknown which blurred the accuracy. Reactions to trauma, cultural frictions and conflicts between the new and old gender norms made the diagnostic process difficult. The patient-doctor encounter comprised misconceptions, and social roles in the meetings were sometimes confused. GPs based their judgement mainly on clinical intuition and the established classification of depressive disorders was discussed. Tools for management and adequate action were diffuse.

**Conclusion:**

Dialogue about patients' illness narratives and social context are crucial. There is a need for tools for multicultural, general practice care in the depressive spectrum. It is also essential to be aware of GPs' own conceptions in order to avoid stereotypes and not to under- or overestimate the occurrence of depressive symptoms

## Background

Depression is a common cause of ill health all over the world [1]. Many depressed patients attend primary health care [2], and minority-group patients often see general practitioners (GPs) for depressive symptoms [3]. The diagnosis and classification of depression is based on the presence of a number of mainly psychiatric symptoms [4,5].

Over the years, however, classification criteria of depression have been discussed among scientists and the peril of under or over-diagnosis of depressive states is addressed [6-8]. The classification was earlier based on aetiology and depression was categorized into "reactive" (exogenous) and "endogenous" depression [9]. The latter included depression as an expression of biological causes and factors from inside while reactive depression comprehended the depressive consequence of an earlier trauma or hardships in life [10]. Since 1980 the aetiological claims have disappeared and the classification of depression now focuses on two dimensions of symptoms, polarity and degree of severity during at least two weeks (DSM-IV and ICD-10).

These classification systems have been criticized as not taking contexts into consideration and the risk of medicalization of "normal" sadness after loss or failures is pointed out [9,10].

Symptoms and reactions to trauma are expressed and experienced differently in different cultures [11,12] and can also vary between men and women [13-15]. These disparities hamper the option of identifying a depressive state in men and women and in patients from different cultures [16-18].

Screening instruments and national guidelines for depression like HAD (Hospital Anxiety and Depression Scale) and NICE (National Institute for Health and Clinical Excellence) are repeatedly suggested by authorities [19,20] and the GPs are quite often blamed for not following guidelines correctly and not recognizing the depressive patient [21-23]. However, primary care differs from specialist psychiatric care. Problems in the primary care setting are often presented on the crossroads between somatic, psychological and social symptom presentations and it is often difficult to apply genuine psychiatric diagnostic systems in primary care [24,25]. Neither diagnostic tools nor guidelines are adapted to specific cultural contexts which make them poorly applicable in clinical practice [17,25].

Studies on the GPs' own struggles in the endeavour to assess a depressive state correctly illustrate that many GPs consider personal qualities and experiences, including those of gender, to be more significant than academic education and professional literature [2,26,27]. The GPs seem to question the validity of diagnostic tools [28] and also questionnaires are doubted as the use of these kinds of instruments might impede the human element of the consultation [29].

Minority groups are known to have poorer health and hurdles appear in the access to health care compared to majority groups [30]. Communication between the foreign-speaking persons and health-care personnel is also known to be difficult and the patients' cultural background, their ideas about health and concern often differ from the ideas of the personnel [31]. This cultural diversity is not always focused on in the patient- doctor encounter which can increase communication problems and complicate the recognition of mental illness [32].

Many immigrants have settled in Sweden during the last decades and people from about 200 countries are living in Sweden today [33]. The most frequent countries of origin of the newly-arrived foreigners besides other Nordic countries, are Iraq, Poland and Somalia [33]. During the last five years many immigrants also from former Yugoslavia, Afghanistan and Iran have arrived in Sweden. Many settlers still have families left behind in their native country and worries about relatives at home are common. In the new country immigrants often live under uncertain circumstances as a minority group [16].

The aim of this study was to explore and analyse how GPs think and deliberate when seeing and treating patients exclusively from foreign countries and who display potential depressive features.

## Methods

Qualitative research may serve as a bridge between theoretical and practical knowledge [34] and interviews in focus groups have been seen as a suitable way to receive information from many informants about a specific topic [35]. Data that are generated from focus groups are also a mixture of personal and collective narratives that are formed by local norms and beliefs in participants' lives [36].

All 18 GPs, even trainees, working at two Primary Health Centres with many immigrants living in the catchment area in Umeå, in Northern Sweden, were invited to focus-group interviews. Immigrants were mainly of Somali, Iraqi and Iranian extraction but people from many other countries also regularly visited the Health Centres. All doctors accepted to participate but three GPs were unable to join in (practical reasons) but the participating 15 GPs represented a broad variation in age, gender and clinical experience. Seven GPs were women and eight were men. Three had been specialists in General practice/Family medicine for more than 30 years, five between 10–20 years, three less than 10 years and two were trainees. Four doctors were also born and brought up outside Sweden. The interviews were carried through at the Health Centres.

One of the GPs could not participate in the group interview because of an urgent practical reason and she was later individually interviewed. Furthermore, one of the GPs who attended a group interview expressed special experiences of other cultures and he was also individually interviewed afterwards. Fourteen GPs participated in the focus-group sessions (two focus- groups with one group session each).

Both the individual and group interviews lasted 1–1.5 hours and were audio taped. One of the authors (BM), both psychiatrist and GP, acted as a moderator and another author (AL) was observer. The individual interviews were conducted by AL. All the interviews took place in May and June, 2008.

The aim of the study was initially presented and the opening question of all interviews was: *Please tell us about your thoughts and reflections when seeing a patient from a foreign culture and who presents possible depressive symptoms?*

This primary query by the moderator gave initially different individual answers and it was followed by further exploration of issues in the field of interest. The group discussions were gradually reinforced by more targeted questions and the participants were encouraged to be as detailed as possible in their comments in order to receive manifold observations. All the GPs were also prompted to participate actively.

The interviews were transcribed verbatim and carefully read through. Afterwards, two of the authors (BM, AL) coded all the texts individually as codes, subcategories and categories according to Qualitative Content Analysis [37]. The preliminary codes were compared and discussed among all researchers. Only minor discrepancies were found and the coding was considered until agreement was reached. The formulated categories and subcategories included factors concerning patients' background, patient-doctor encounter and reflections about help and treatment. The underlying meaning throughout the codes, subcategories and categories was interpreted during the process and the interpretation was formulated as three themes, threads of meaning throughout the subcategories (Table 1).

**Table 1:** The three themes with categories

Themes	Categories
Realizing the background	Experienced hardships
	Fundamentals changed
	Cultural ideas and clashes

Struggling for clarity	Misunderstandings in communication
	Doctor's role in transition
	Image of depression questioned

Optimizing management	Measures to be followed
	Assignment questioned

The theoretical approach in this study is based on a conceptual understanding that scientific knowledge is always "situated" [38]. "Situated knowledge" implies that knowledge must always be interpreted in relation to the society and the context in which it is created. The data and analysis of this study are situated and are intended to be relevant in particular to the context under study and for this research question.

According to the regional research ethics committee and Swedish legislation the study did not need a formal ethical approval. However the study was carried through according to general ethical procedures like voluntariness, possibility to break the participation at any time and written and oral information.

## Results

Three themes; "**realizing the background**", "**struggling for clarity**" and "**optimizing management**" were elaborated from the GPs' narratives. The themes partly reflect a sequence of the presented descriptions; about the previous history, the actual encounter and thoughts on follow-up and treatment.

### Realizing the background

The patients' previous history was crucial. A thorough background was rarely known but the GPs stated that "***experienced hardships***", "***fundamentals changed" ***and "***cultural ideas and clashes" ***were categories that illustrated important spheres for the origin of depressive features.

Before leaving their native country many persons had been exposed to traumatic events, (***experienced hardships***), which have been influential in the creation of the depressive moods. The GPs presented several examples of patients with distressing experiences leading to post-traumatic suffering. Occasionally these life events could be explicitly expressed and estimated. Frequently however the patients' earlier history remained mainly unknown and many of the GPs were not acquainted with the patients' former experiences. This vagueness influenced the doctor- patient encounter and many possible decisive factors were not touched upon.

*"It can be experiences of war, torture or anything else, trauma we have no idea about. ... "(F 6); "... women could have been exposed to sexual violence. You can suspect that there is something" (M 12);" .... It takes several years to discover what lies behind..." (M8); "...the Swedes, maybe, find it especially difficult to understand how stigmatizing this can be...do I dare to ask about it?" (F7);"... you begin to ask an immigrant when they arrive in Sweden and you reflect on the background..." (F7)*.

The impact of leaving the old and adapting to a new country (***fundamentals changed***) was stressed. Their status as an exile meant sorrow and distress; the loss of their country in itself, the characteristic smells and sounds that used to be heard implied an existential loss and feelings of alienation. A longing to go "home" was expressed and patients' worries about relatives and friends in the home countries were revealed in several narratives.

Migration resulted quite regularly in loss of status. The previous, self- evident social position was questioned. The power in gender relations could be changed and differ from the norms in the old country. The GPs realized that dramatic changes in status and social circumstances could cause tensions and conflicts leading to anxiety and depressive expressions.

*".. Patients' ideas are that the husband protects the family; he is working... But here he is nobody...it affects even men ..." (M 4);"Male immigrants get more easily depressed if their wives manage life better" (M 1, F 10);"Male immigrants' gender role is challenged here... "(F 6); "Many of the women live very isolated lives at home; it is amazing that not many more of them are depressed "(F 15); "...Women who have always been at home, taken care of children and cooked the meals are stressed to go out and study Swedish..."(M 3)*.

Views on health are determined by explanations of health and illness and the presentation of patients' symptoms was influenced by the cultural background (***cultural concepts and clashes***). Depression as a disease entity is missing in some cultures and it was difficult to talk about something "non-existent". To be depressed was in some societies the same as being mad and just to mention depression was regarded as an insult. It was difficult for the GP to relate to the collective culture among some groups, for example among Romani people. Romani people presented imprecise troubles as a group, difficult for the GP to interpret according to his/her habitual agenda. The GPs expressed obstacles in individualizing such problems.

The GPs conveyed opinions of being too uninformed about foreign cultural and religious norms and notions on health. Any views were taken from media and occasional narratives implying a risk for stereotypes and generalizations. No professional support on cultural issues was at hand.

*" ..In some cultures it is just "normal" to be sad " (M 4);" The Swedes accept the concept of depression much more today and you do not talk about pulling one's socks up" (F 7); "It is easy to be prejudiced against women from the Middle East... this is depression... things we have imagined or heard from media" (F 10);" I try not to think, she is from Iraq and she must have difficulties. I listen to her somatising depression ... it is difficult when you see her the first time, maybe next time you can label it as depression" (M 12); "The Romani people seldom come alone. They see us in groups...how to separate the problems of this patient from what the others have to say" (M 1)*.

### Struggling for clarity

The patient-doctor encounter embraces several dilemmas. "***Misunderstandings in communication"***, "***doctor's role in transition***" and "***image of depression questioned***" were categories that encompass areas of indistinctness.

The diagnosis of depression is mainly based on symptoms and no objective tests exist. Verbal and nonverbal communications are the central elements in the interaction and accordingly ***misunderstandings in communication ***were frequently reported. The presence of a professional interpreter could enhance understanding while children and relatives as interpreters more often hinder comprehension. The triad-interaction (a patient, an interpreter and a doctor) was time-consuming and frictions in communication were common.

Patterns of nonverbal communication vary between countries. A resigned posture or avoidance of eye contact was difficult to evaluate. Did these signs indicate depression or just "normal" cultural behaviour? As the concept of depression does not exist or is repressed in some cultures the GPs reported hurdles in topics of emotions. To use the word depression could be regarded as shameful.

*"I feel handicapped, nuances can disappear in communication"(M3); " The problem can also be the interpreter... she did not want the interpreter to know ... because of religion" (F5); "People from Iraq or Afghanistan seem to be quite serious. I do not know if they are always like that, if it is a cultural feature ..., women with covering clothes and a hidjab... it is difficult to interpret" (F 15); "You choose your words in order not to hurt anybody's feelings. It is very difficult if you communicate through an interpreter" (F10)*.

The patients sometimes challenged the gender or the working style of the GP (***doctor's role in transition***). Many patients were unfamiliar with a patient-centred working style, a more authoritative orientation by the doctor was asked for. Patients were not used to express their own opinions in the consultation and a GP's request like "*what do you think yourself"*, was rather regarded as unskilled doctoring than as a sign of interest.

A patient-doctor encounter with different sexes implied impediments. A male patient originating from a hierarchic society with a traditional gender order was puzzled when meeting a female doctor. To carry out physical examinations of patients of a different sex also often created setbacks.

*"Our tradition is to negotiate. It is unfamiliar to many immigrants... you shall know and give an answer" (F 6); "A man, who does not want to shake hands with me as a woman gives a signal of a possible troublesome contact "(F13); "... I try to be more resolute with hesitating men and I want to show I am the doctor..." (F15); ..."or a woman who does not want to be examined or touched by a man...if they have pain and you are not allowed to examine...you can't manage it ..." (M 12); "In many cultures people are told that men have more knowledge and power .. and maybe an immigrant does not trust a female doctor" (F7)*.

The GPs regarded the conceivable manifestations of depression as unclear and typical diagnostic criteria of depression were hard to apply (***image of depression questioned***).

Various bodily symptoms or excessive use of alcohol were looked upon as potential signs of depression. Pain could indicate depression but cultural variations in the expressions of pain blurred the diagnostic validity. The picture of pain could also vary among patients from the same cultural sphere and the level of education influenced the symptom presentations. Patients with a low level of education more often displayed non-distinctive symptoms and the manifestations were tricky to interpret. No difference in gender appearances was mentioned but women attended health care more frequently.

The GP's main assets in the management of potential depressive states were the clinical experience, a sense of intuition while criteria according to diagnostic manuals gave little backing. Screening-instruments, sometimes recommended and used, were not regarded as suitable for these patients. Thus, the diagnostic power was low.

The GPs depicted the patients often as clearly unhappy or sad and their predicament was mainly regarded as a response to life stressors and the exile status. The disease entity of depression was questioned.

"They have bodily symptoms..." (M1); " I have not noticed any gender difference in expressions of depression" (F15); "Men seek less help for depression than women, immigrant men as well as Swedish men take alcohol or something else" (F6);

"The scale of HAD does not fit for these groups" (M 2); "The diagnosis 'depression' is very much based on feelings (M 8).. clinical experience (M1)..and intuition" (F15);

*"I wonder if our diagnosing of symptoms as depression is a way of making it easier for us instead of managing the real situation" (M3); "Many patients are suffering and sad, maybe not what we really mean by depression..."(F9); "Once I met a patient who went home to his native country and he felt much better..."(M8)*.

### Optimizing management

The GPs contemplated what kind of treatment and support could be offered (***measures to be followed***) and also considered the task of the doctor in these cross-cultural encounters. Feelings of powerlessness emerged among the GPs (***assignment questioned***).

Depressive symptom presentations based on hard life conditions were intricate to accommodate (***measures to be followed***). Difficulties in verbal communication restricted the exchange of ideas, yet a family- oriented dialogue in a cognitive style was regarded as a possible way to organize and care for the unhappy or aching patient.

The use of anti- depressive medication was questioned and most GPs were hesitant to prescribe these drugs. The outcome of a drug delivery was often negative; the effect on Swedish patients was considered more positive. Yet, the GPs prescribed occasionally anti-depressives even if compliance was poor and the consequences were doubtful. A kind of helplessness and lack of powerful means to alleviate the patients' distress was evident. The peril of medicalization of human stresses was discussed.

An offer of a sick- leave certificate was one way to mitigate a difficult situation temporarily. Yet, it could be hard to set bounds to patients' demands for sick- leave certificates. It was difficult to strike a balance to between patients' demands, their vulnerable life situations and the GPs' own beliefs and willingness to accept. Consensus was not easy to attain.

"It is very complicated to understand, there are both bodily symptoms and social complications" (M2);" but how can a person get cognitive behaviour therapy, without language skills?" (F9);" It would be important to take care of the whole family" (M4);

*"...I think I understand how they feel but they do not always understand what I mean... (F15)"; "...We can't manage their problems with a lot of pills... A person's feeling bad because of the life situation ..." (M2); "...Drugs are the only thing you can give quite promptly" (F6)*.

*"For a woman from Iraq who has eight children and has always been at home, is sick-leave certification a solution for a severe social situation?" (M2);" And how to come to a solution in 30 minutes?" (M3)*.

The task of the GP in these consultations was sometimes questioned and reflected on by the interviewees (**assignment questioned**). The task of the doctor was often to face existential problems as much as medical deficiencies and it was not self-evident that the problems could be solved within health care. Political actions and support groups in areas with many immigrants would be more effective in relieving human problems.

*"We can't solve all the problems people present at a surgery...other implements are necessary; people from the same country can be engaged and give information about hurdles..."(M4));" An unemployed immigrant woman with many children who cannot speak Swedish, living in economic hardships ... it is a normal reaction...what can we do?"(F10)*.

## Discussion

Our study showed that GPs had difficulties in managing patients from other cultures who expressed potential depressive features. Knowledge about patients' earlier years was often unknown. Cultural and gender frictions in the patient – doctor encounter and the process of diagnosing and managing comprised other important uncertainties. Feelings of powerlessness emerged quite often among the GPs.

The traditional diagnostic tools and guidelines for depression seemed to offer limited support for the GPs in helping the patients. Immigrants' early traumatic experiences and severe life circumstances could lead to distress and GPs considered often their presenting symptoms as reasonable reactions to earlier life events and tangible life conditions rather than signs of a depressive disorder. The prescribing of drugs or recommending sick-leave certificates for psychosocial problems felt unsatisfactory. Indecisiveness forced the GPs to ad hoc solutions.

The GPs had acquired most of their knowledge and clinical experiences by studying Western medical literature and in the encounters mainly seen patients from their own culture. Seeing patients with other backgrounds implies however a modification [16,31].

### Disorder versus normal reaction

GPs considered depression as a socially more accepted entity in Sweden today while at the same time the diagnostic credibility has not been increased. This acknowledgment of depression as a medical entity could be reflected in a tendency that individuals sometimes stated themselves as depressed rather than tried to manage their exposed condition. GPs also apprehended that depressive symptoms among patients from other countries besides being responses to life circumstances also illustrates frictions between an old and a new gender order [2,26] and it is more seldom that low-spirited features constitute a major depression.

Many GPs considered the relevance of the governing classification of depression that focuses on the two dimensions of symptoms, polarity and degree of severity (DSM-IV and ICD-10). The older nosology that divides depressions into "reactive" (exogenous) and "endogenous" depression was quite unanimously preferred [9]. This previous standpoint has also been discussed in a recent editorial on depression by Cole et al. [7]. The authors write: "*Our current nosologies remain as a working hypothesis and have no greater validity than the definitions of depression that existed when Kendell wrote in 1976*." [7].

The doctors were ambivalent to giving drugs to patients with possible reactive depressive features and the risk of medicalization of human suffering was discussed. Studies have also stressed that classification of depression without taking into consideration the context of symptoms might lead to the overturning of sadness into disease [6,10]. Suffering caused by trauma and life circumstances need primarily practical measures – drugs were more unwillingly prescribed.

According to the local guidelines two symptoms, depressed mood and a decreased interest in everyday duties are the first steps in the depressive diagnostic procedure. A screening instrument, HAD -scale [19], is also recommended by a specialist board of the local County council.

The GPs found it difficult however to apply these symptoms as the basis for the judgement of an existing depression. The depressive mood was difficult to estimate owing to uncertainties in communication, both verbal and non-verbal. The backing of an interpreter enhanced the clarity but a verbal support did not compensate for the linguistic deficiency, also reported in other studies [31]. A diminished interest in everyday duties was also difficult to estimate as the patients were in the process of more or less successfully adopting to a new country and new cultural norms. Instead of focusing on depressed mood and decreased interest in everyday duties the GPs paid attention to presented bodily symptoms, often seen as reactions to anxiety and hardships. The GPs very rarely used the screening instrument (HAD) as linguistic difficulties and problems in fully understanding the answers created interpreting difficulties.

The GPs based most of their diagnosis on a general clinical experience including gut feeling and common sense. These considerations correspond to earlier observations that clinical familiarity and intuition are important tools in the diagnostic process of depressive illnesses [2,26,27].

All GPs conveyed a request for more knowledge on variations of depressive signs when seeing patients from other cultures than their own. Several researchers have also highlighted that depression guidelines are not adapted to primary care or to multi-cultural environment and to the importance of seeing psychiatric disorders as context-bound [11,25].

A recent study on immigrant patients in USA is also in line with the GPs' attitudes to the nosology of depression. Depressive patients in primary health care divided causes of their depression as internal or external [39]. The patients were doubtful about drug treatment when external explanations to the depression were dominating. Drugs were however preferred if the causes according to the patients' opinion were internal.

### GPs' dilemma

The GPs reported conflicts between the cultural norms of the patient and the norms of Swedish society; gender norms were especially reported. These clashes could sometimes lead to various annoyances, suffering and despair. In these consultations GPs had difficulties in striking a balance between the demands of society, the possibilities of supporting the patients and their own norms.

GPs' narratives included several gendered and generalized descriptions of "the suffering woman", who was a woman from the Middle East with another cultural gender order. The GPs thoughts about these women, living their lives at home and caring for many children illustrate the cultural and gender conflicts which in turn exemplify that gender stereotypical thinking could lead to an improper diagnosis of depression. A potential to misdirect diagnosis and treatment of depression due to construction of cultural stereotypes of women from the Middle East is discussed even in other studies [40]. However, the GPs in our study seemed to be aware of the risks of stereotyped preconceptions. They expressed a fear of misdiagnosis and mistreatment of depression and difficulties in striking a balance between the individual, cultural and social levels in health care.

A requirement for sick-leave by the patient also included a dilemma. Sick absenteeism could lead to more isolation but with few alternatives to provide help and support a sick-certificate could compensate for the GPs' dilemma and helplessness [41].

In the Western world the traditional, dominating form of masculinity emphasises that men should be strong rather than weak [42]. This socially accepted gender norm can be related to men's denial of depressive feelings, a preference for alcohol as a means of self-care and less help-seeking for depression [43]. The application of the Western norms to all men and ignoring a possible vulnerability of men from other countries as a subordinate, minority population [44] illustrates another quandary in the management of male patients with roots in other cultures.

According to Western medicine a patient-centred working style and mutual understanding are crucial in a good patient -doctor encounter [45]. A continuous dialogue and a family orientation were regarded as potential ways to care for a patient with depressive symptoms even if time was restricted for this measure. GPs with a Swedish background especially emphasised the difficulties in asking about the patients' background[33]. This could be interpreted as a lack of personal experiences of severe trauma leading to barriers in the caring process [32]. Studies on communication with patients from other countries show that factors like dialogue about patients' illness experiences, situational awareness about cultural differences and physicians self-awareness and adaptability to individualized communication are seen to be crucial in culturally competent communication [46],

The GPs in our study also noticed cultural dissimilarities in patients' willingness to be involved in health care [46]. It was also evident that gender norms influenced the patient-doctor encounter and patients' expectations of a more authoritative doctor challenged the GPs. A female doctor meeting a male patient was provoked by his not shaking hands in deference to religion [47]. It was reported as tempting to change working style and be more authoritative as a way of bringing back control. The GPs however, remained uncomfortable in such procedure [48].

### Strengths and limitations of the study

A qualitative dynamic form of analysis that stays close to data was searched for and a content analysis method is a suitable method [35,37]. The analysis of verbal data summarizes the informal content of the interviews and discussions. The method allows handling of large amounts of data in a systematic way, compressing texts into a limited number of categories, using defined rules of coding.

The data were mainly collected by focus-group interviews. It is a method suitable for collection of research data involving individuals from a population that is appropriate to the particular interests of the researcher. The data collected in groups is formed by GPs own cultural and gendered backgrounds but is at the same time produced in the interaction between the participants [37]. The fact that four of the GPs (two in group one and two in group two) were born and brought up outside Sweden had an impact on the discussions. Relevant and interesting topics were brought into the talks by these GPs. At the same time, their presence might result in an inhibition of some other less influential GP's viewpoints.

The material obtained from the group interviews was rich and illustrative. The successive two individual interviews generated some further information but these talk mainly confirmed data already received. The study included 15 experienced GPs with professional experiences overstepping 220 years.

The fact that the interviews were carried out by colleagues to the informants made it more likely to increase their willingness to communicate and may have had impact on the broad scope and richness of the material [49]. On the other hand, the interviewer's experience of primary care may have created a shared blindness and may have influenced the dialogue, interpretations and the results. There is an urge among GPs to do their work in the "right" way, and also a fear of failure according to professional standards. This tends to be replicated in research when both the interviewer and the interviewed are GPs. The informants may experience the interview as a test of their interest and clinical experience, especially when carried out in a group setting [36,50]. The loss of this possibly hidden agenda among GPs could be a weakness of the study, yet we believe that the openness in the discussions and the voluntariness to participate guaranteed a considerable level of credibility. Preliminary results of the analysis were also discussed with some of the participating GPs at the end of the coding procedure. The feedback supported our findings and increased the trustworthiness of the analysis [38].

## Conclusion

The study highlights the complicated nature of the diagnosis and management of depressive symptoms when GPs see patients from other cultures. The medical model with its protocols and guidelines for the diagnosis and treatment of depression does not fit with the everyday experience of these GPs. Depressive symptoms, which are difficult to be sure of are often regarded as signs of hardships and reactions to earlier trauma or a severe life situation. The question is whether the GPs have the possibility of managing these losses and shortcomings. The diagnosis and management of depressive symptoms are also formed by the gender of patient and doctor, cultural norms as well as verbal and nonverbal communication.

There is a need for tools for multicultural mental health primary care in order to promote communication and improve clinical diagnosis and management of patients' depressive symptoms. At the same time a continuous dialogue and GPs' situational awareness as well as an awareness of own conceptions in order to avoid stereotypes are crucial in order to diminish risks for under- or overestimation of the depressive symptoms and to offer equal care for all. The multicultural appearances of depressive symptoms are a challenge for GPs but to improve the life circumstances which lead to a depressed mood and suffering is a challenge for society.

## Competing interests

The authors declare that they have no competing interests.

## Authors' contributions

AL, AH and BM designed the study. AL and BM were responsible for the data collection. AL carried out all analyses with support from BM and AH. AL wrote a draft of the manuscript which BM and AH read and commented upon. All authors have read and approved the final manuscript.

## Pre-publication history

The pre-publication history for this paper can be accessed here:


